# Decoding Dysregulation of DNA Methylation in Epigenetics of Idiopathic Pulmonary Fibrosis

**DOI:** 10.1155/carj/2927505

**Published:** 2026-04-22

**Authors:** Guorao Wu, Xinhong Tang, Yinjian Zhou, Yi Wang, Danlei Yang, Lei Zhang

**Affiliations:** ^1^ Department of Pulmonary and Critical Care Medicine, NHC Key Laboratory of Respiratory Diseases, Tongji Hospital, Tongji Medical College, Huazhong University of Science and Technology, 1095 Jiefang Avenue, Wuhan, 430030, China, hust.edu.cn

**Keywords:** DNA methylation, epithelial cells, fibroblasts, idiopathic pulmonary fibrosis, macrophages

## Abstract

Idiopathic pulmonary fibrosis (IPF), the continuously advancing and frequently lethal idiopathic interstitial pneumonia, is pathologically characterized by abnormal deposition of extracellular matrix constituents, leading to progressive pulmonary scarring. Although substantial research efforts have been devoted to elucidating its pathogenesis, the etiology, molecular mechanisms, and effective treatment strategies for IPF remain inadequately characterized, contributing to a median postdiagnostic survival period of merely three to five years. Epigenetic modifications, particularly DNA methylation, have emerged as a potential missing link between the genetic background and environmental risk factors contributing to the pathogenesis of fibrotic disorders, including IPF. DNA methylation entails the attachment of a methyl group to the cytosine residue, frequently occurring at CpG dinucleotides, which are concentrated in clusters known as CpG islands and orchestrates the target gene expression. In this review, we will explore the mechanism of DNA methylation and provide insights into recent advancements concerning its role in major profibrotic cells within lung tissues during the progression of IPF, and by which, we aim to generate valuable evidence that can facilitate the advancement of antifibrotic therapies for clinical applications.

## 1. Introduction

Idiopathic pulmonary fibrosis (IPF), the predominant type of idiopathic interstitial pneumonia, represents a progressively and chronic advancing fibrotic lung disease with unknown etiology [1]. IPF imposes a substantial burden on affected individuals, often resulting in irreversible lung fibrosis, chronic respiratory failure, and ultimately, hypoxemia‐associated mortality [2]. In the absence of effective treatment, IPF patients face a median survival period of less than 5 years [3]. With the rising global burden from IPF, there is a critical demand for deeper insights into its pathogenesis and the development of targeted strategies to curb fibrosis progression.

As a cornerstone epigenetic mechanism, DNA methylation induces stable, epigenetic modifications in gene expression and chromosomal function independent of DNA sequence variation. It entails the methyl group of S‐adenosylmethionine (SAM) being added to the fifth carbon (C5) or the fourth nitrogen (N4) of cytosine bases under the catalysis of the family of DNA methyltransferases (DNMTs) [4]. As a crucial epigenetic mechanism, DNA methylation participates in essential biological processes such as suppressing retroviral sequences, regulating tissue‐specific gene activity, controlling genomic imprinting, and mediating X chromosome inactivation [5–9]. Advances in methylation profiling technologies have shed light on its significance in various diseases, including lung fibrosis [10–12].

In this review, we aim to meticulously examine the existing body of knowledge concerning the engagement of DNA methylation of major profibrotic cells during the pathogenesis of pulmonary fibrosis. We seek to advance the mechanistic understanding of IPF pathogenesis and propose novel therapeutic strategies based on epigenetic insights.

## 2. The Basic Pathogenesis of IPF

IPF is characterized by the abnormal buildup of extracellular matrix (ECM) within lung interstitium. Pathogenesis of IPF is related to the complex relationships and interactions among genetic susceptibility, environmental exposure, and cellular dysfunction [13]. Recurrent minor injuries to the aging alveolar epithelial tissue result in cellular aging and programmed cell death [14]. Subsequently, epithelial cells release the profibrotic growth factors and cytokines, boosting abnormal epithelial–mesenchymal crosstalk, the recruitment, and subsequent activation of myofibroblasts, which exhibit heightened synthetic and contractile properties [15]. Additionally, alveolar epithelial cell death attracts inflammatory cells like macrophages, and recruited cells secrete plenty of cytokines and chemokines to create a profibrotic microenvironment for injury repair [16]. This progressive sequence of events leads to an excessive deposition of ECM components, including fibronectin, collagens, proteoglycans, and hyaluronic acid, thereby triggering a cascade of lung tissue remodeling [17, 18]. Consequently, an excessive accumulation of scar tissue develops within the lung tissues.

### 2.1. Overview of DNA Methylation

Unlike genetics, which studies sequence‐based inheritance, epigenetics investigates heritable phenotypic changes that occur without alterations to the DNA sequence [19]. Key epigenetic processes—including DNA methylation, diverse histone modifications, and nucleosome repositioning—collectively enable precise regulation of gene activity. These synergistic components ensure the precise regulation of gene activity and the establishment of stable gene silencing. Of these mechanisms, DNA methylation, an integral epigenetic process, has garnered extensive and widespread research attention.

DNA methylation occurs through enzyme‐catalyzed transfer of methyl groups to cytosine residues, generating 5‐methylcytosine or N4‐methylcytosine as the modified products [20], respectively. In theory, any cytosine have the potential to be methylated, methylated cytosine is predominantly found in large clusters known as CpG islands (CGIs), which are primarily located in the promoter or first exon region of genes [21]. CGIs are typically defined as regions that exhibit the following characteristics: (1) a length exceeding 200 base pairs, (2) a C + G content surpassing 50%, and (3) a CpG observed/expected ratio greater than 0.6, despite alternative definitions are occasionally employed. The human genome features around 25,000 CGIs (excluding repetitive elements), approximately 75% of which span fewer than 850 base pairs. Importantly, around 60 to 70% of human genes include a CGI within their promoter regions. Nevertheless, it is crucial to note that the mere presence of CGI does not inherently govern gene regulation. The mechanisms underlying DNA methylation encompass three fundamental components: writers, erasers, and readers, which refer to the enzymes responsible for establishing, removing, and recognizing DNA methylation, respectively.

#### 2.1.1. Writers

In mammalian systems, DNMTs serve as the primary enzymes responsible for DNA methylation. These enzymes facilitate the transfer of a methyl group from SAM to the carbon of cytosine residues, primarily within the C5 of CpG dinucleotide contexts. The DNMT family encompasses DNMT1, DNMT2, DNMT3a, DNMT3b, and DNMT3L. DNMT2 and DNMT3L were previously considered to lack cytosine methyltransferase activity [22]. DNMT1, classified as a maintenance DNMT, primarily functions to restore the parental DNA methylation pattern during replication, while members of the DNMT3 family serves as de novo methyltransferases, responsible for methyl group transfer onto unmethylated DNA regions [23]. Regardless of the gene expression pattern, DNMT3a and DNMT3b are extremely similar in structure and function involved in DNA methylation [24].

#### 2.1.2. Erasers

DNA demethylation mechanisms can be categorized as passive or active processes. Passive demethylation mainly takes place in proliferating cells following DNMT1 inhibition or dysfunction, leading to gradual loss of methylation patterns [25]. In contrast, active demethylation directly modifies 5 mC through a series of chemical reactions, mainly including deamination and oxidation pathways. The chemically modified bases are subsequently replaced via the base excision repair (BER) pathway. Notably, cytidine deamination is facilitated by AID/APOBEC, whereas oxidation of methylated cytosine is mainly carried out by TET enzymes, such as Tet1, Tet2, and Tet3 [26, 27]. Notably, the last step of this process is carried out by the BER pathway: thymine DNA glycosylase recognizes and then removes the modified cytosine, which is then replaced with unmodified cytosine, thereby achieving complete DNA demethylation [28].

#### 2.1.3. Readers

Gene expression can be suppressed by DNA methylation through direct interference with transcription factor binding to DNA. Furthermore, there exists a class of proteins in cells that specifically recognize methylated DNA sequences and participate in transcriptional regulation by binding to methylation sites [29]. Currently, it is known that three major protein families are responsible for recognizing DNA methylation signals: the MBD family, the UHRF family, and the zinc finger protein family [30]. Among these families, the MBD was the first to be identified and have been extensively studied. This highly conserved MBD protein is expressed ubiquitously and interacts with the nucleosome remodeling and histone deacetylation (NuRD) complex [31]. MBD family consists of methyl‐CpG‐binding protein 2 (MeCP2) and MBD1–6. Except for MBD3 and MBD5‐6, the majority of MBD family members are capable of binding methylated DNA directly and modulating downstream transcriptional activity.

### 2.2. The Implication of DNA Methylation in Pulmonary Fibrosis

**FIGURE 1:**
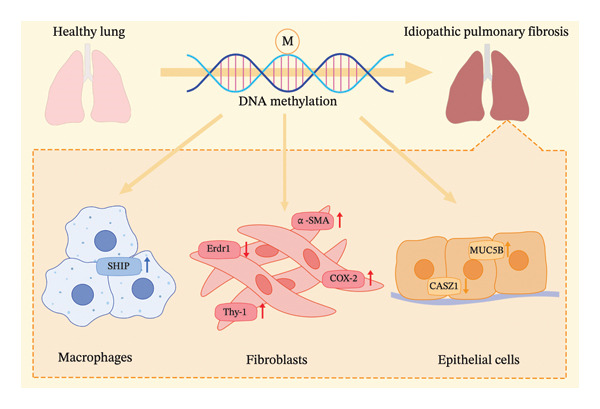
DNA methylation changes of specific genes in profibrotic cells during the development of pulmonary fibrosis. MUC5B: mucin 5 subtype B; Ship: SH2‐containing inositol 5′‐phosphatase; α‐SMA: α‐smooth muscle actin; cyclooxygenase‐2: COX‐2; ↑: hypermethylation.

Both hypermethylation and hypomethylation contribute significantly to IPF pathogenesis. It should be emphasized, however, that methylation patterns are highly cell‐type specific and often differ substantially between bulk lung tissue and individual cellular populations (Figure 1).

### 2.3. Global DNA Methylation and IPF

Early research efforts focused on mapping the epigenomic methylation patterns across the entire genome within pulmonary samples of IPF patients, seeking to define the disease’s characteristic methylation signature [12, 32]. In one prominent study by Sanders et al., DNA methylation microarray analysis was employed to systematically map modifications of methylation patterns in IPF lungs. A research indicated that, despite observed alterations in the expression of DNMTs (such as DNMT3a and DNMT3b), no significant difference in global DNA methylation levels was detected between IPF and normal tissues [12]. Nevertheless, the study identified 870 differentially methylated genes from IPF samples—406 hypomethylated and 464 hypermethylated compared with controls. Further validation of selected genes, including CLDN5, ZNF467, TP53INP1, and DDAH1, confirmed correlations between methylation status and expression: hypermethylation of CLDN5 and ZNF467 was associated with reduced expression, whereas hypomethylation of TP53INP1 and DDAH1 corresponded to increased expression [12]. These findings provided early strong support for the involvement of DNA methylation about IPF pathogenesis. Similarly, another study reported 625 differentially methylated CGIs in IPF samples, with 91.2% resided in intronic, exonic, or intergenic regions, while mere 8.8% in promoter areas [32]. However, these studies were limited by small sample sizes, warranting further investigations. Subsequently, a study utilizing comprehensive high‐throughput methylation arrays examined genome‐wide methylation and gene expression patterns in a larger cohort [33]. It revealed more extensive methylation changes in IPF lungs, predominantly occurring outside CGIs [34]. Later integrated bioinformatics analyses supported this observation and indicated that methylation sites associated with IPF were enriched in similar biological pathways [35, 36]. Collectively, these studies offer compelling evidence about the involvement of epigenetic dysregulation, particularly DNA methylation, contributing to the development of IPF.

It should be noted that while the studies summarized above focused on bulk lung tissue, DNA methylation alterations are both gene specific and cell‐type specific, and their functional impacts during IPF progression may vary accordingly. Furthermore, methylation profile of the promoter region CGIs can mediate gene transcription straightly, which means CpG methylation in specific genes across various cell types remains a vital and highly complex biological process in multiple levels.

### 2.4. DNA Methylation and Lung Epithelial Cells

Mucin 5 subtype B (MUC5B) is a key gel‐forming mucin present in lung tissue, playing vital roles in mucociliary clearance and host defense, which is secreted by both proximal submucosal glands as well as those lining the distal airways [37, 38]. IPF is characterized by aberrant accumulation of MUC5B protein within the epithelial lining of respiratory bronchioles and honeycomb cysts—sites intimately linked to active fibrotic remodeling [39, 40]. Initial reports indicated a significant correlation between the common polymorphism rs35705950 in the MUC5B promoter and improved survival in IPF patients [41, 42], which has driven extensive research into the mechanisms regulating MUC5B expression.

Research by Britney A. Helling and colleagues revealed an important regulatory domain from the promoter variant of MUC5B that encompasses a well‐conserved FOXA2 binding site. This region exhibits differential methylation patterns correlated with IPF pathogenesis, MUC5B expression levels, and the rs35705950 genotype. Research demonstrates that elevated methylation levels correlate with advancing IPF, the risk allele T, and upregulation of MUC5B expression, thereby accelerating disease development [43]. The investigation also indicated that transcription factors including HOXA9, ZBTB7A, and STAT3 might participate in regulating MUC5B expression and IPF pathogenesis through mechanisms separate from the promoter polymorphism [43].

A key protein within the shelterin complex is Protection of Telomeres 1 (POT1), which plays a vital role in telomere protection and length regulation by binding telomeric single‐stranded DNA and promoting telomerase recruitment [44]. Gene sequencing revealed the presence of POT1 site mutations in patients with IPF family inheritance, accompanied by telomere deletions, DNA damage, and premature aging [45]. Researches have suggested that cellular aging is an essential factor for the occurrence of progressing fibrosis in the lungs [14, 46]. Through Mendelian randomization analysis, it was found that short telomere length is highly correlated with the occurrence of IPF [47]. Recent work by Mengkun Shi et al. revealed that epigenetic silencing via DNA methylation—mediated by DNMT and MeCP2—suppresses POT1 expression, thereby inducing alveolar type 2 (AT2) cell senescence and accelerating fibrotic remodeling [48]. The specific mechanism by which DNA methylation accelerates cellular aging is currently unknown. Undoubtedly, POT1 is a key link and promising therapeutic target for treating fibrotic lung.

Another noteworthy gene in pulmonary fibrosis research is Castor zinc finger 1 (CASZ1), a gene regulatory protein featuring zinc finger motifs involved in blood vessel formation and cardiac fibrosis [49]. Methyl‐eQTL analysis of IPF and donor tissues identified four transregulatory methylation sites near CASZ1 that may modulate its expression [33]. CASZ1 primarily facilitates vascular formation and morphological development through its interaction with intronic sequences in EGFL7—a secreted angiogenic factor that associates with ECM components and potentially influences Notch signaling pathways. [50]. CASZ1’s involvement in ECM and Notch signaling pathways in IPF cells highlights its importance for further investigation in pulmonary fibrosis.

### 2.5. DNA Methylation and Fibroblasts

IPF is characterized by myofibroblast‐driven collagen overproduction, culminating in irreversible lung architecture damage, respiratory function decline, and patient death [13].

As a potent profibrotic mediator, transforming growth factor‐β1 (TGF‐β1) plays a central role in activating fibroblasts. Miguel Negreros et al. investigated the impacts of transient and sustained TGF‐β1 exposure on gene expression patterns and epigenetic DNA methylation from normal lung and IPF fibroblasts [51, 52]. Remarkably, the fibroblasts from IPF patients exhibited substantial methylation changes, predominantly hypermethylation [53]. Phenotypically, IPF fibroblasts displayed distinct characteristics compared to non‐fibrotic lungs, including elevated α‐smooth muscle actin (α‐SMA) levels [54] alongside suppressed expression of key antifibrotic mediators. Among the downregulated genes are cyclooxygenase‐2 (COX‐2) [55], Thy‐1 Cell Surface Antigen (Thy‐1) [56], Caveolin‐1 (Cav‐1) [57], C‐X‐C motif chemokine ligand 10(CXCL10) [58], and superoxide dismutase 2 (SOD2) [59]. Emerging evidence indicates that aberrant epigenetic mechanisms significantly contribute to both the induction of profibrotic pathways and the specific suppression of antifibrotic genes within fibroblast populations [10].

A correlation was observed between the methylation status of CGIs within the α‐SMA promoter, as well as its expression level [60]. Through gel shift and chromatin immunoprecipitation (ChIP) analyses, it was shown that MeCP2 directly interacts with the promoter of the α‐SMA gene [61]. Interestingly, contrary to previous reports suggesting MeCP2 primarily functions in gene silencing via specific methylated CGIs, their results indicated that a significant proportion of MeCP2‐bound promoters are associated with actively expressed genes, revealing a dual regulatory role for MeCP2 in transcriptional regulation [61, 62]. In addition to regulating α‐SMA during fibroblast‐to‐myofibroblast transition, MeCP2 was shown to downregulate Wnt inhibitory factor‐1 (WIF1) expression, an antagonist of the Wnt pathway, thereby facilitating fibroblast activation [63]. Another reader of DNA methylation, MBD2, was also implicated in myofibroblast formation during the progression of IPF. Notably, our published data demonstrated a significant upregulation of MBD2 in myofibroblasts, depending on the TGFβR1/Smad3 signaling pathway. MBD2 is found to specifically interact with methylated CpG sites in the Erdr1 promoter, suppressing Erdr1 and amplifying TGF‐β/Smad signaling, thereby driving myofibroblast differentiation and aggravating pulmonary fibrosis [64].

Abnormal fibroblasts isolated from IPF and systemic sclerosis (SSc) patients exhibited reduced capacity to produce COX‐2, as well as its downstream antifibrotic factors, prostaglandin E2 (PGE2) [65]. Iona C. Evans et al. elucidated that the decreased mRNA expression of COX‐2 in IPF fibroblasts is regulated by epigenetic mechanisms. Although the methylation pattern of COX‐2 did not emerge striking variation in fibrotic and normal fibroblasts according to bisulfite sequencing and methylation microarrays, further analysis identified c8orf4, a transcriptional regulator, as a mediator of COX‐2 expression through its binding to the proximal promoter in fibroblasts [66]. Collectively, the function of the COX‐2/PGE2 axis in fibrotic lung fibroblasts is impaired, which can be traced back to the hypermethylation of the transcriptional regulatory factor c8orf4; this epigenetic event indirectly leads to the silencing of antifibrotic signaling pathways.

Bone morphogenetic proteins (BMPs), as signaling molecules within the TGF‐β superfamily, regulate critical processes in IPF, including fibroblast invasion and migration capabilities. It is observed that DNA methylation–induced downregulation of BMP binding endothelial regulator (BMPER) promoter activity and expression results in dysregulation of the BMP signaling pathway; however, the precise molecular mechanisms underlying this epigenetic regulation remain to be fully elucidated [67].

Thy‐1 is an important protein involved in tissue fibrosis, particularly within the lungs, making it an ideal candidate for assessing methylation levels in related studies [68, 69]. In lung tissues from IPF patients, Thy‐1 expression is silenced due to promoter hypermethylation, highlighting the pivotal effect of DNA methylation between driving fibroblast dysfunction and promoting pulmonary fibrotic progression [56].

SOD2 is a mitochondrial antioxidant enzyme critical for redox homeostasis, and mitochondrial oxidative damage, driven by SOD2 dysfunction, is recognized as a pivotal contributor to IPF progression [70]. Notably, recent studies demonstrate that TGF‐β‐induced downregulation of SOD2 in lung fibroblasts is mediated by DNA methylation, highlighting the significance of epigenetic modification​ in mitochondrial redox imbalance and fibrotic remodeling [59]. However, the underlying mechanisms linking these processes remain incompletely elucidated and warrant further investigation.

### 2.6. DNA Methylation and Macrophages

Macrophages serve as the initial defense in the airway innate immune response against pathogens [71, 72]. The phenotype and number of macrophages are thought to be critically involved in the pathobiology of IPF [73, 74]. Peter McErlean and colleagues employed Illumina EPIC arrays to analyze alveolar macrophages from healthy and IPF donors, demonstrating epigenome alterations linked to AM development and function in fibrotic lungs [11]. Their research detected 11 differentially methylated positions and 49 differentially methylated regions in IPF alveolar macrophages relative to healthy controls. These differential methylation events involved genes associated with lipid metabolism (LPCAT1) and glucose metabolism (PFKFB3) [11]. Intriguingly, reducing DNA methylation levels through knockout of DNMT3b expression significantly facilitated alternative macrophage polarization and enhanced phenotypes associated with experimental pulmonary fibrosis [75].

Previous research has extensively established the important contributory role of macrophages, particularly the alternatively activated (M2) subtype, in the initiation and advancement of lung fibrosis [76–78]. In recent years, our laboratory has focused on investigating the impact of DNA methylation on pulmonary fibrosis. Our research has uncovered disruptions between DNA methylation profiles and MBD2 expression in both patient IPF and experimental bleomycin models. Correspondingly, mice lacking MBD2 specifically in macrophages exhibited protection from bleomycin‐induced pulmonary fibrosis. MBD2 deficiency markedly declined TGF‐β levels and diminished M2 macrophage accumulation in pulmonary tissues. Mechanistic studies revealed that MBD2 specifically interacts with the SH2‐containing inositol 5′‐phosphatase (Ship) gene promoter in macrophages, thereby suppresses Ship, leading to amplified PI3K/Akt pathway activity and driving the cells toward an M2‐polarized state. Consequently, the intratracheal administration of nanoparticles carrying MBD2 siRNA provided significant protection against bleomycin‐induced pulmonary fibrosis in mice [79].

A recent study from Mou et al. validated through animal experiments that regulating interferon expression can reduce MeCP2 overexpression and effectively reduce macrophage polarization [80]. Collectively, DNA methylation is fundamentally implicated in both the initiation and advancement of IPF, as well as provides effective strategies for the IPF clinical treatment strategies.

### 2.7. Therapy Perspectives

Advances in our understanding of the biological basis of epigenetics, particularly DNA methylation, have unveiled novel therapeutic avenues. As principal effector cells in advanced lung fibrosis, fibroblasts are instrumental in the development of fibroblastic foci. Given the association between DNA methylation and fibroblast function, targeting the regulation of specific gene methylation in fibroblasts holds immense potential for the treatment of lung fibrosis.

Recent research highlights the advantages of DNA methylation–based therapies. DNMT inhibitors, particularly 5‐Azacytidine (5‐AZA) and its derivative 5‐aza‐2′‐deoxycytidine (5‐AZAdc), represent a class of epigenetic modulators that have been extensively investigated and employed in clinical practice [81]. These agents have received FDA approval for therapeutic application in myelodysplastic syndromes and acute myeloid leukemia [81, 82]. The aforementioned evidence revealed the significance of hypermethylated genes including Thy‐1 and COX‐2 in modulating fibroblast behavior and advancing fibrotic progression. Numerous studies have investigated the effects of DNMT inhibitors and other demethylating drugs on lung fibrosis, both in vivo and in vitro.

Experimental evidence indicates that 5‐AZA and Zebularine (a cytidine nucleoside analog) can reinstate PGE2‐mediated suppression of collagen production and fibroblast proliferation [83, 84]. Similarly, research demonstrates that 5‐AZAdc reduces collagen levels and reinstates apoptotic sensitivity in IPF fibroblasts via demethylation of c8orf4, a transcriptional regulator of COX‐2 [66]. Given the involvement of TGF‐β/BMP signaling in pulmonary fibrogenesis, studies further reveal that 5‐AZA downregulates BMPER expression, diminishing ECM production and migratory activity in IPF fibroblasts, with comparable effects observed in murine fibrosis models. In the work by Zheyi Xiang et al., treatment with 5‐AZA, the DNMT inhibitor, would cause diminished α‐SMA levels in human fibroblasts, accompanied by reduced MeCP2‐binding activity [85].

## 3. Conclusion

In conclusion, this review provides a comprehensive summary of the core pathological mechanisms involved in IPF and DNA methylation (Figure 1). The pathogenesis of fibrotic lung diseases involves a sophisticated network of interactions among diverse cell types and signaling cascades, each subject to modulation by epigenetic mechanisms. Additionally, interactions between DNA methylation and other epigenetic mechanisms, such as histone modifications and noncoding RNA, are also implicated in IPF [33]. Recent advances suggest that cell‐free DNA methylation patterns may work as biomarkers to IPF diagnosis or differential diagnosis [86, 87], which could facilitate more accurate clinical assessment. Moreover, emerging evidence on methylation‐based epigenetic clocks and their correlation with IPF disease severity offers a promising avenue for further exploration [88]. The persistent therapeutic challenges in IPF underscore the necessity of continued investigation into epigenetic mechanisms involved in fibrogenesis, which may yield innovative approaches to halt or reverse pulmonary fibrosis progression.

## Author Contributions

Conceptualization, Lei Zhang and Danlei Yang; writing–original draft preparation, Guorao Wu and Xinhong Tang; supervision, Yi Wang and Yinjian Zhou.

## Funding

Our work was supported by the Key Project of Tongji Hospital Research Fund (2024A18).

## Disclosure

All authors gave their approval for the final manuscript to be published.

## Consent

The authors have nothing to report.

## Conflicts of Interest

The authors declare no conflicts of interest.

## Supporting Information

This study was prepared with reference to the Strengthening the Reporting of Observational Studies in Epidemiology using Mendelian randomization (STROBE‐MR) statement. It is important to note that as a summary‐level review, many items in the STROBE‐MR checklist—particularly those pertaining to original study design, participant recruitment, and ethical approvals—are not directly applicable to the present work. We have, however, fully addressed the items in the “Other Information” section of the checklist, including statements on funding, data availability, and conflicts of interest (provided on page 15 of this manuscript). Adherence to relevant reporting guidelines aims to enhance the transparency and interpretability of our findings.

## Supporting information


**Supporting Information** Additional supporting information can be found online in the Supporting Information section.

## Data Availability

The data presented in this study are available on request from the corresponding authors.
